# Data on mass spectrometry based identification of allergens from sunflower (*Helianthus annuus* L*.*) pollen proteome

**DOI:** 10.1016/j.dib.2016.03.023

**Published:** 2016-03-12

**Authors:** Nandini Ghosh, Gaurab Sircar, Bodhisattwa Saha, Naren Pandey, Swati Gupta Bhattacharya

**Affiliations:** a Division of Plant Biology, Bose Institute, Kolkata, West Bengal, India; b Department of Allergy and Asthma, Belle Vue Clinic, Kolkata, India

## Abstract

Allergy is a type of abnormal immune reactions, which is triggered by environmental antigens or allergens and mediated by IgE antibodies. Now-a-days mass spectrometry is the method of choice for allergen identification based on homology searching. Here, we provide the mass spectrometry dataset associated with our previously published research article on identification of sunflower pollen allergens (Ghosh et al., 2015 [1]). In this study allergenicity of sunflower (*Helianthus annuus*) pollen grains were primarily investigated by clinical studies followed by detailed immunobiochemical and immunoproteomic analyses. The mass spectrometry data for the identification of allergens were deposited to ProteomeXchange Consortium via PRIDE partner repository with the dataset identifier PXD002397.

**Specifications Table**

**Table:** 

Subject area	*Biology*
More specific subject area	*Mass spectrometry*-*based identification of allergen*
Type of data	*Figure*, *Table*
How data was acquired	*MALDI*-*TOF*/*TOF* (*Autoflex II*, *Bruker Daltonics*, *Germany*) *and LC*-*ESI qTOF* (*maXis Impact, Bruker Daltonics, Germany*) *analysis of in*-*gel digested proteins*
Data format	*Analyzed mass spectrometry data*
Experimental factors	*Total pollen proteome of Helianthus annuus was confronted with pooled sera of sunflower pollen*-*sensitized patients. Allergenic proteins were excised from 2D gel*, *and in*-*gel digested spots were identified using a combination of two different mass spectrometric techniques* (*MALDI TOF*/*TOF and LC*-*ESI qTOF*) *and searching the MS*/*MS data against NCBInr database and EST library of H. annuus.*
Experimental features	*Trypsin digested IgE reactive proteins were subjected to MALDI*-*TOF*/*TOF. The raw MS*/*MS spectra were initially searched against NCBInr database. Unmatched peptides with low significance score were further subjected to similarity searches against the H. annuus EST database Unmatched proteins, after MALDI-TOF*/*TOF, were subjected to LC*-*ESI qTOF for better peak resolution, maximum signal, and maximum coverage. They were loaded on nano-LC-ESI MS/MS. Similarly, MS/MS spectra were searched against NCBInr and EST library of H. annuus. Finally, FASTA sequences of matched EST clones were analyzed by BLASTx algorithm.*
Data source location	*Bose Institute*, *Kolkata*, *India*
Data accessibility	*Data available within this article and accessible at ProteomeXchange Consortium via PRIDE partner repository with the dataset identifier PRIDE*: *PXD002397*.

## Value of the data

• Seven allergenic proteins were identified from sunflower pollen using a combination of two ionization methods and two different databases.
• Proteins were identified from a plant without having any prior information on its genome sequence. Such strategy can also be effectively applied for other wildly grown weed species for which no genome information is available.
• This data will be helpful for further studies on these allergens such as recombinant expression, epitope mapping, study of cross-reactivity, etc.

## 1 Data

Sunflower pollen is an important source of inhalant allergens. In this data article we are sharing the mass spectrometry data for identification of seven allergens from sunflower pollen. This dataset is associated with the article “**Search for allergens from the pollen proteome of sunflower (*****Helianthus annuus*** **L.): A major sensitizer for respiratory allergy patients**” [1].

## 2 Experimental design, materials and methods

**Fig. 1:**
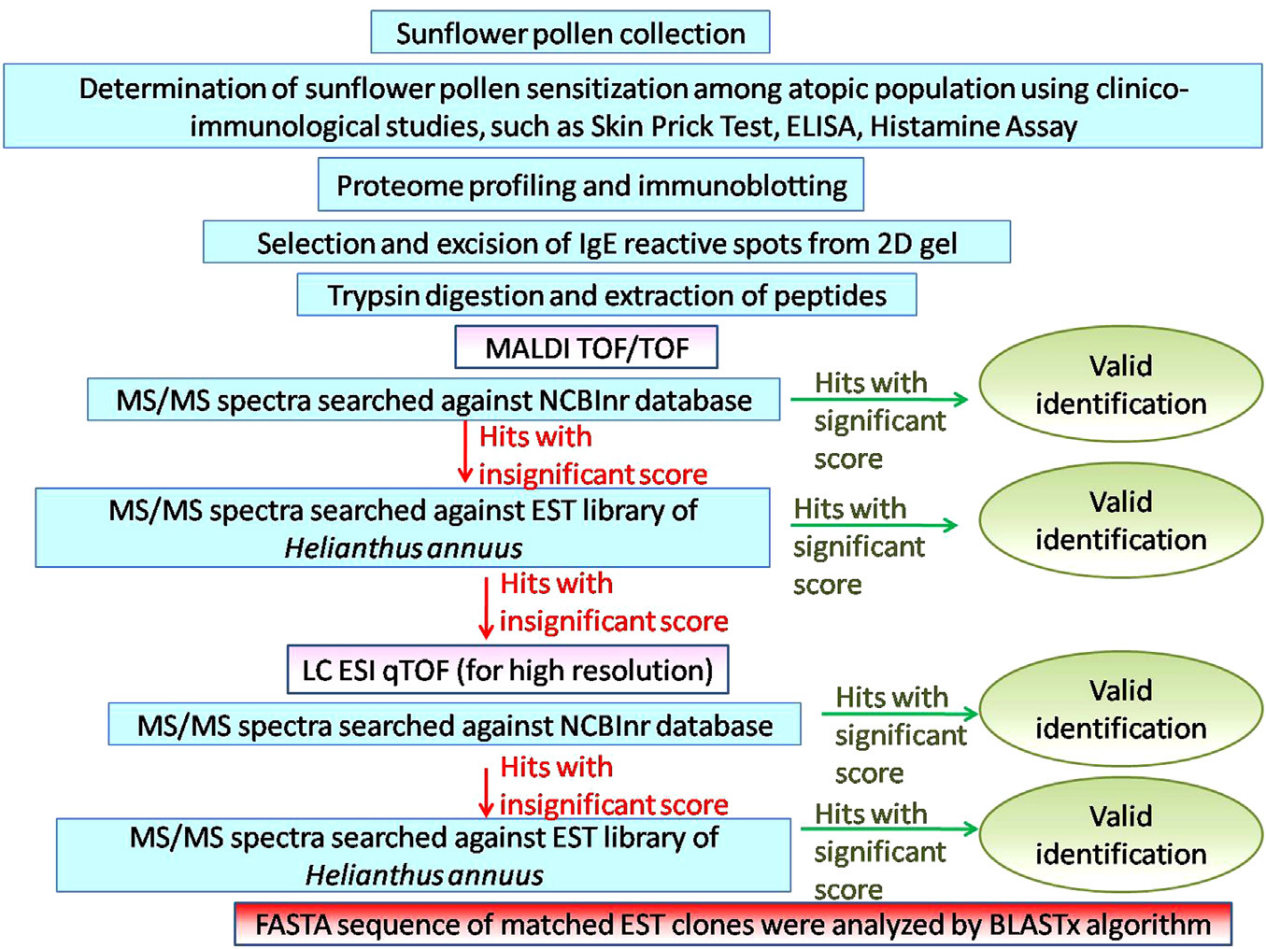
Experimental Workflow.

Using bottom-up proteomic approach we have previously identified sunflower pollen allergens [1]. The experimental workflow (illustrated in Fig. 1) was designed to identify allergenic pollen proteins of sunflower. First, clinico-immunological tests were performed to understand the prevalence of sensitivity towards sunflower pollen among the atopic population. Sera from selected sunflower positive patients were used as the probe to detect the IgE-reactive proteins from two-dimensional electrophoretic separated proteome of sunflower pollen. Finally, these allergens were identified by mass-spectrometry.

### 2.1 Pollen sampling and protein extraction

Pure pollen grains of *H. annuus* were collected from mature anthers of the fresh flowers growing around the city during their peak flowering period (April to the first week of July of 2012–2014). Total pollen protein was extracted from 1 g of pollen in 20 ml of 0.1 M phosphate buffer (PB), pH 7.2 and used for serological studies. For proteomic analysis, total protein was extracted using trichloro-acetic acid (TCA)-acetone protocol following the method described earlier [1].

### 2.2 Patient selection and clinical studies

Pollen allergic patients, visiting Mediland Diagnostics, Kolkata were tested with antigenic extract of sunflower pollen grains. Patients with positive cutaneous response against sunflower pollen antigen were selected, and 5 ml of peripheral blood were collected for immunological studies. The patient group in our study was represented by 20 individuals sensitive to sunflower pollen, while the control group included six non-atopic subjects. The total IgE, specific IgE and released histamine in these sera were quantified. This study protocol was approved by the human ethics committee of Bose Institute and Mediland Diagnostic Clinic, Kolkata. Informed written consents were obtained from patients and non-allergic volunteers for participation in the study. In the case of minors, informed written consents were obtained from their guardians.

### 2.3 2 Dimensional (2D) gel electrophoresis and 2D immunoblot

2D electrophoresis was performed following the protocol earlier described [2] with minor modification. Around 120 µg of protein was reconstituted in 125 μl rehydration buffer containing 0.75% IPG (pH 3–10 linear) buffer (v/v) (GE Healthcare), 25 mM DTT and traces of bromophenol blue. The sample was applied to 7 cm IPG strip (pH 3–10 Linear) in a re-swelling tray and left overnight at room temperature for rehydration. Separation of proteins in the first dimension was carried out in Ettan IPGphor 3 isoelectric focusing system (GE Lifescience) as per manufacturer׳s instructions. The second dimension separation was performed in miniVE Vertical Electrophoresis System (GE Healthcare).

For immunoblot, Proteins from 2D gel were transferred onto polyvinylidene difluoride (PVDF) membrane (GE Lifesciences) by semi-dry transfer method [3]. 1:10 diluted pooled sera (sera of twenty sunflower pollen sensitized patients׳) were used as primary antibody, while 1:1000 diluted monoclonal anti-human IgE alkaline phosphatase conjugate served as secondary antibody. Healthy serum pool was taken as negative control. Blots were developed with nitro-blue tetrazolium-5-bromo-4-chloro-3′-indolylphosphate (NBT-BCIP) (Sigma). Images of immuno-blots were acquired in Bio-Rad Versa Doc (Bio-Rad Laboratories) system and analyzed by Quantity One® software (version 4.6.3, Bio-Rad Laboratories). We considered only those sero-reactive spots with high signal (intensity value greater than 2).

### 2.4 Sample preparation for mass spectrometry

For mass spectrometry (MALDI TOF/TOF and LC-ESI qTOF), spots from 2D gel corresponding to the IgE reactive spots on 2D blot, were excised and subjected to in-gel trypsin digestion following the protocol as described by Shevchenko et al. [4] with slight modifications. Briefly, the gel pieces were destained with ethanol in 50 mM ammonium bicarbonate (pH 8.0) (1:1 v/v) and Acetonitrile (ACN). Reduction and alkylation was done with 10 mM DTT and 55 mM iodoacetamide respectively. Digestion was carried out in 12.5 ng/µl modified sequencing grade Trypsin Gold (Promega) at 37 °C for 16 h. Tryptic fragments were eluted from gel pieces by vigorous vortexing in extraction buffer containing 3% TFA and 30% ACN. The final volume of the sample was reduced up to 10 times in Speed Vac (Thermo Fischer). Approximately, 1.5 μl of peptide digests were mixed with 5 volumes of 0.5 mg/ml α-cyano-4-hydroxycinnamic acid (CHCA) matrix solution (Bruker Daltonics), spotted on MTP 384 ground steel target plate (Bruker Daltonics) and air dried. For LC-ESI experiments the solvent was completely evaporated and the peptides were dissolved in a suitable volume of 2% ACN containing 0.1% formic acid. This reconstituted sample was then mixed with solvent A used for loading onto LC-column.

### 2.5 MALDI-TOF/TOF analysis

Mass spectra of trypsin-digested proteins were obtained in Autoflex II MALDI-TOF/TOF (Bruker Daltonics). Mass spectra were recorded in linear mode equipped with a pulsed N_2_ laser (*λ*=337 nm, 50 Hz) at 54% power in positive ion mode. After MS spectra acquisition, the instrument was switched to LIFT mode. The MS/MS spectra of top ten peptides with the highest intensity were recorded by fragmentation of these peptides using LID (laser induced dissociation). MS/MS spectra were acquired with a minimum of 4000 and a maximum of 8000 laser shots using the instrument calibration file. Spectra baseline subtraction, smoothing [5] and centroiding were performed in Flex Analysis software v3.0 (Bruker Daltonics).

### 2.6 LC-ESI qTOF analysis

All the MS and MS/MS experiments for peptide identification were performed using a maXis impact™ high-resolution qTOF mass spectrometer (Bruker Daltonics) equipped with a Captive Spray (Bruker Michrom) electrospray ionization platform. Separation was performed on a Dionex PepMap C18 column (250 mm×75 µm, 3 µm particle). The flow rate was set at 300 nL/min. The mobile phases A and B were 0.1% formic acid in water and 0.1% formic acid in 80% ACN, respectively. Positive ions (charge state +1, +2, +3) were generated by the electrospray ionization captive source. The following source settings were used for all subsequent data collection: Drying gas (nitrogen): 3 L/min, Dry temperature: 150 °C, Capillary voltage: start at 1500–1700 V, decrease in 50 V steps until signal drops and add 300 V, End plate offset: 0 V.

Survey scans were acquired within a range from 200 to 2000 m/z. The spectrometer sequentially conducted MS/MS (in CID chamber) on the precursor ions (+2 and +3 charge state, excluding +1) detected in the full scan in data independent manner. MS/MS scans were conducted within a range from 25 to 2000 m/z. All MS and MS/MS raw data were acquired in .mgf format using Bruker׳s ProteinScape™ software (version 3.0).

### 2.7 Database search and allergen identification

The raw MS/MS spectra processed using MS Biotools™ 3.2 (Bruker Daltonics) was used as input to an in-house MASCOT search engine version 2.2 using the following criteria: minimum signal-to-noise ratio: 20; peak density filter: 5 peaks per 200 Da and maximum number of peaks: 20. Searches were conducted with the following settings: one missed cleavage, *P*<0.05 as significance threshold, the mass tolerance of precursor and fragment ions: 0.5 Da and 1.2 Da, respectively for MALDI TOF/TOF whereas 40 ppm and 100 ppm respectively for LC-ESI qTOF, carbamidomethylation of cysteine as fixed modification, methionine oxidation as variable modification, peptide charge:+1 for MALDI TOF/TOF and +2, +3, +4 for LC-ESI qTOF. Spectra were initially searched against NCBInr database; however, unmatched peptides with low significance score were further subjected to similarity searches against the *H. annuus* EST database (downloaded from NCBI on December 2015) containing 134474 entries. FASTA sequences of respective EST clones were analyzed by BLASTx algorithm, which searched for any signature sequence in the translated query to identify the protein. The mass spectrometry data may be accessed from PRIDE (http://www.ebi.ac.uk/pride/archive/). Supplementary Table 1 [S1.Table] represents concise form of MS/MS search results against different databases and BLASTx analysis.

## Footnotes

Appendix A Supplementary data associated with this article can be found in the online version at doi:10.1016/j.dib.2016.03.023.
